# Impact of vitamin A supplementation on infant and childhood mortality

**DOI:** 10.1186/1471-2458-11-S3-S20

**Published:** 2011-04-13

**Authors:** Aamer Imdad, Mohammad Yawar Yakoob, Christopher Sudfeld, Batool A Haider, Robert E Black, Zulfiqar A Bhutta

**Affiliations:** 1 Division of Women & Child Health, The Aga Khan University, Karachi, Pakistan; 2 Department of International Health, Johns Hopkins Bloomberg School of Public Health, Baltimore, Maryland, USA

## Abstract

**Introduction:**

Vitamin A is important for the integrity and regeneration of respiratory and gastrointestinal epithelia and is involved in regulating human immune function. It has been shown previously that vitamin A has a preventive effect on all-cause and disease specific mortality in children under five. The purpose of this paper was to get a point estimate of efficacy of vitamin A supplementation in reducing cause specific mortality by using Child Health Epidemiology Reference Group (CHERG) guidelines.

**Methods:**

A literature search was done on PubMed, Cochrane Library and WHO regional data bases using various free and Mesh terms for vitamin A and mortality. Data were abstracted into standardized forms and quality of studies was assessed according to standardized guidelines. Pooled estimates were generated for preventive effect of vitamin A supplementation on all-cause and disease specific mortality of diarrhea, measles, pneumonia, meningitis and sepsis. We did a subgroup analysis for vitamin A supplementation in neonates, infants 1-6 months and children aged 6-59 months. In this paper we have focused on estimation of efficacy of vitamin A supplementation in children 6-59 months of age. Results for neonatal vitamin A supplementation have been presented, however no recommendations are made as more evidence on it would be available soon.

**Results:**

There were 21 studies evaluating preventive effect of vitamin A supplementation in community settings which reported all-cause mortality. Twelve of these also reported cause specific mortality for diarrhea and pneumonia and six reported measles specific mortality. Combined results from six studies showed that neonatal vitamin A supplementation reduced all-cause mortality by 12 % [Relative risk (RR) 0.88; 95 % confidence interval (CI) 0.79-0.98]. There was no effect of vitamin A supplementation in reducing all-cause mortality in infants 1-6 months of age [RR 1.05; 95 % CI 0.88-1.26]. Pooled results for preventive vitamin A supplementation showed that it reduced all-cause mortality by 25% [RR 0.75; 95 % CI 0.64-0.88] in children 6-59 months of age. Vitamin A supplementation also reduced diarrhea specific mortality by 30% [RR 0.70; 95 % CI 0.58-0.86] in children 6-59 months. This effect has been recommended for inclusion in the Lives Saved Tool. Vitamin A supplementation had no effect on measles [RR 0.71, 95% CI: 0.43-1.16], meningitis [RR 0.73, 95% CI: 0.22-2.48] and pneumonia [RR 0.94, 95% CI: 0.67-1.30] specific mortality.

**Conclusion:**

Preventive vitamin A supplementation reduces all-cause and diarrhea specific mortality in children 6-59 months of age in community settings in developing countries.

## Introduction

Vitamin A includes a group of fat soluble compounds that are involved in growth and differentiation of various body cells. These include cells of the respiratory epithelium, gastrointestinal tract, retina and immune system [1]. Vitamin A has been termed as an anti-infectious vitamin because of its role in regulating human immune function. Early studies in animals and humans revealed an association between vitamin A deficiency and increased susceptibility to infections [1,2]. Its deficiency makes humans, especially infants, vulnerable to diseases of eye, respiratory and gastrointestinal tract [3]. These observations encouraged evaluation of effect of vitamin A supplementation on morbidity and mortality in randomized controlled trials (RCTs).

Past reviews have evaluated the impact of vitamin A supplementation on infant and childhood mortality. A meta-analysis combining 6 community-based RCTs showed a reduction of 30% [Relative risk (RR) 0.70 95% confidence interval (CI): 0.62-0.79] in all-cause mortality and a reduction of 39% [RR 0.61 95% CI: 0.50-0.76] in deaths from diarrheal disease in pre-school children [4]. There was no effect on deaths related to respiratory diseases. Another systematic review including 10 RCTs with 8 included in a meta-analysis, showed a significant reduction of 23% [RR 0.77; 95% CI 0.68-0.88) in all-cause mortality among vitamin A supplemented children compared to controls in children 6-60 months of age [5]. The review reported a significant reduction in mortality due to diarrheal disease [RR 0.71; 95% CI: 0.57-0.88) and measles [RR = 0.46; 95% CI: 0.22-0.98] and a non-significant effect on deaths attributed to respiratory disease [RR = 0.94; 95% CI: 0.63-1.42]. A review of vitamin A supplementation in childhood and pregnancy published in Lancet Under-nutrition Series by Gogia et al. [6] also showed a significant reduction in all-cause mortality in pre-school children [RR = 0.77, 95% CI: 0.63-0.95].

Reviews have also been conducted for neonatal vitamin A supplementation. An analysis for neonatal vitamin A supplementation by Haider et al. showed a reduction of 20% [RR 0.80; 95 % CI: 0.66-0.96] in infant mortality at 6 months of age; the results for mortality at 12 months of age were, however, not statistically significant [RR 0.90; 95% CI: 0.61 to 1.32] [7]. Another meta-analysis published in British Medical Journal evaluating effect of neonatal vitamin A supplementation on infant mortality showed that neonatal vitamin A supplementation had no effect in reducing all-cause mortality at 12 months of age [RR 0.92; 95% CI: 0.75 to 1.12] [8].

We evaluated all the available evidence on impact of vitamin A supplementation in reducing infant and childhood mortality. This exercise is a part of series of efforts to estimate efficacy of an intervention for input to Lives Saved Tool (LiST) model [9]. An intervention is currently included in the LiST if there is evidence that it reduces mortality among children less than five years of age, either directly or indirectly. The process of generating a point estimate for efficacy of an intervention, in reducing disease specific deaths or a risk factor, involve qualitative evaluation of available evidence according to Grading of Recommendations, Assessment, Development and Evaluation (GRADE) criteria [10] and quantitative measure according to Child Health Epidemiology Reference Group (CHERG) rules [9]. For more details of the review methods, the adapted GRADE approach or the LiST model see the methods section and other articles in this supplement.

## Methods

### Searching

We systematically reviewed all published literature to identify studies evaluating preventive effect of vitamin A supplementation on infant and childhood mortality. We searched PubMed, Cochrane Library, and all World Health Organization Regional Databases and included publications in any language. Following search strategy was used on PubMed: [“Children” OR “Child” OR “Infant” OR infan* OR “neonate” OR neonat* OR “newborn” OR “Preschool”] AND [“vitamin A” OR "retinol" OR “retino*”]. The search was limited to “clinical trial” and “humans”. Last date of search was 3^rd^ March 2010. We scanned the titles and abstracts of the trials identified to exclude those that were obviously irrelevant, retrieved the full text of the remaining trials, and identified relevant articles. We also reviewed the reference lists of identified articles, existing reviews and meta-analyses and looked for studies that were not picked up in the main search. Authors were contacted for any additional data, if required.

### Inclusion/exclusion criteria

All the included studies were randomized or cluster randomized controlled trials addressing vitamin A supplementation in neonates (0-28 days of life) or children < 5years of age. Trial authors were contacted if the study population included some participants who were not eligible for this review (e.g., children over 5 years), and requested disaggregated data. If such data were not available, studies were included if the majority of participants met the inclusion criteria. If this could not be determined and the participants met the inclusion criteria on average, then these trials were included. The comparison group in all the included studies was either supplemented with a placebo or observed as controls. In studies where vitamin A was supplemented to both mother and infant we considered only those pairs (of mother/infant) where mother received placebo or no intervention. Studies were included if they reported data on: all-cause mortality and/or disease specific mortality of diarrhea, pneumonia, measles, meningitis and sepsis. The cause of death was assigned as defined by the authors in individual studies, mostly on the basis of verbal autopsies. Participants in all the included studies were apparently healthy children in whom prophylactic, synthetic vitamin A supplementation was initiated in neonatal period (<1 month of age) or early childhood (1-59 months of age), We excluded trials conducted on selected subgroups of infants, such as those who were very low birth weight (<1500 g), sick or admitted to hospital, as these have been examined elsewhere [11-14].

### Data abstraction

We abstracted the data of included studies onto a standardized abstraction form [9] for each outcome of interest. We abstracted key variables with regard to the study identifiers and context, study design and limitations, intervention specifics, and outcome effects. The data were entered by two authors and discrepancies were removed if found.

### Validity assessment

Each study was assessed and graded according to the CHERG adaptation of the GRADE technique [9,10]. This method of assessment is based on scores allocated to individual studies on the basis of study design, quality of methods, relevance to the objectives of the review and consistency across studies. Studies received an initial score of high if a randomized or cluster randomized trial and then the grade was decreased for each study design limitation, if applicable. In addition, studies reporting an intent-to-treat analysis or with statistically significant strong levels of association (>80% reduction) receive 1-2 grade increases. Each study was assigned a quality grade of “high” “moderate” “low” or “very low” on the basis of strengths and limitations of study. Any study with a final grade of very low was excluded from the analysis [9].

### Quantitative data synthesis

We generated meta-analyses for impact of vitamin A supplementation on all-cause and disease specific mortality, where data was available from more than one study. Relative risk was self calculated if not stated in the study, with the following preference order for the denominator: numbers with definite outcome known till completion of intervention period, number randomized and stated child years. For cluster randomized trials, we used the stated cluster adjusted relative risk and 95% confidence interval, irrespective of the method used. We adjusted the results for cluster design if not stated in the study. This was done by inflating the standard error of calculated relative risk by square root of design effect [15]. The value of design effect was taken as stated in the study or was inferred from the previous review by Beaton et al [5]. Pooled estimates of the evaluated outcome measures were calculated by the generic inverse variance method. This method is a common and simple version of the meta-analysis procedure and is so named because the weight given to each study is chosen to be the inverse of the variance of the effect estimate (i.e. one over the square of its standard error) [15]. In this way larger studies, which have smaller standard errors, are given more weight than smaller studies, which have larger standard errors. This minimizes the imprecision (uncertainty) of the pooled effect estimate. The assessment of statistical heterogeneity among trials was done by visual inspection i.e. the overlap of the confidence intervals among the studies, and by the Chi square (P-value) of heterogeneity in the meta-analyses and I^2^ value. A low P value (less than 0.10) or a large chi-squared statistic relative to its degree of freedom (I^2^ >50 %) was considered as providing evidence of significant heterogeneity. In situations of substantial or high heterogeneity being present, causes were explored by sensitivity analysis and random effects model were preferred for pooled analysis. Although random model is not a substitute for a thorough investigation of heterogeneity, it takes an ‘average’ effect from all the included studies compared to fixed models that take the exact contribution from the individual studies [15]. Results of pooled estimates are described as relative risk (RR) with 95% confidence interval (95% CI). All meta-analyses were conducted using software Review Manager version 5 [16].

We undertook a subgroup analysis for effect of vitamin A supplementation in three age groups i.e. neonates, infants 1-6 months and children 6-59 months of age. This subgroup analysis was based on difference in design and conduct of vitamin A supplementation in neonates compared to older children [17] and going beyond neonatal, on proposed differences in efficacy of vitamin A in infants 1-6 months and children 6-59 months of age as reported previously [18]. We also undertook subgroup analysis according to different geographical regions based on the hypothesis that preventive vitamin A supplementation would have highest impact on mortality in Asia. This premise was based on the observation made in the latest report of WHO on prevalence of vitamin A deficiency (serum retinol <0.70 μmol/l) worldwide [19]. According to this report South-East Asia has the highest burden of biochemical vitamin A deficiency. This subgroup analysis was however only limited to all-cause mortality as we did not have sufficient studies on cause specific mortality to conduct an analysis according to various geographical regions.

We present our results of meta-analyses for all-cause and cause specific mortality in the following theme: pooled results from all the included studies irrespective of age of supplementation, with sub-groups for supplementation in neonates (0-28 days), infants 1-6 months and children 6-59 months of age. To get a point estimate of efficacy of preventive vitamin A supplementation, we summarized the evidence for each outcome including qualitative assessment of ‘overall’ evidence according to GRADE criteria and quantitative measures according to standard guidelines of CHERG group [9]. The qualitative evaluation of the overall (pooled) evidence was based on the volume and consistency of the evidence across studies, the size of pooled relative risk and the strength of the statistical evidence for an association between the intervention and the health outcome as reflected in the p-value. In this way, we generated estimated efficacy of vitamin A supplementation in reducing disease specific mortality for the developing countries in community settings and gave our recommendations for input to Lives Saved Tool (LiST) model [9].

## Results

### Trial flow

**Figure 1:**
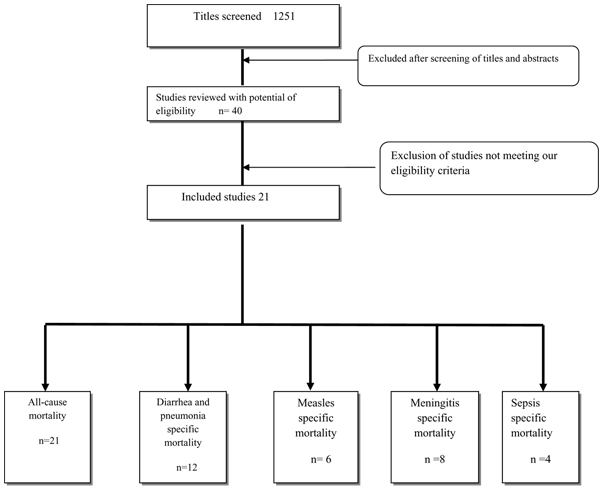
Synthesis of study identification in review of the effects of vitamin A supplementation on infant and childhood mortality

We identified 1251 titles from searches conducted in all databases (Figure 1). After screening the titles and abstracts, 40 studies were initially considered eligible and finally, 21 studies were selected for final data abstraction. We evaluated the impact of preventive vitamin A supplementation on the following outcomes: all-cause mortality, disease specific mortality of diarrhea, pneumonia, measles, meningitis and sepsis.

### Study characteristics

Additional File 1 presents characteristic of included studies. There were 21 randomized controlled trials that addressed preventive vitamin A supplementation in infants or children and reported all-cause mortality [20-40]. Of these, 10 were individual RCTs [28,31-37,39,40], while 10 were cluster randomized trials [20-25,27,29,30,38]. The paper by WHO VAST (Vitamin A Supplementation Treatment) group [26] in Ghana was divided into two studies: VAST survival study (mainly looked at mortality outcomes) and VAST health study (mainly looked at morbidity outcomes). VAST health study was an individual randomized controlled trial while VAST survival study was cluster randomized. Participants in five studies were all neonates [31,35-38], while in nine they were aged 1-59 months [21,22,24,28,30,32,33,39,40]. Seven studies also included children more than five years of age [20,23,25-27,29,34]. Disaggregated data for children < 5 years of age was available from two of these studies and was thus used accordingly [20,23]. For rest of the studies we used the data according to the inclusion criteria. Vitamin A was supplemented in synthetic form in a dose of 50,000 IU to neonates (0-28 days of life), 100,000 IU to infants ( 1-12 months of age) and 200,000 IU to children older than infants according to WHO guidelines [41] except in three studies [21,33,39]. In one of these studies [21], participants received a weekly dose of 8333 IU for 52 weeks, while in other two studies [33,39] it was a dose of 25 000 IU with immunization schedule. In three studies comparison group did not receive a placebo but children were simply observed as controls [20,24,39]. The coverage of intervention was high and it reached > 90 % in fifteen of the included studies [20-23,25-27,31-33,35-38,40]. All the included studies were from developing countries. Additional File 2 presents risk of bias table according to the Cochrane handbook.

### Quantitative data synthesis

#### All-cause mortality

**Figure 2:**
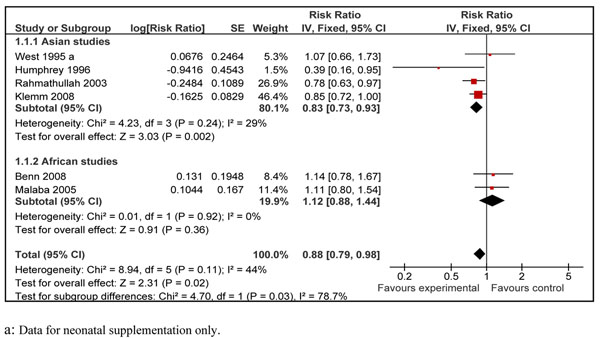
Forest plot for preventive effect of vitamin A supplemenation on all-cause mortality at six months of age with subgroup analysis according to geographical region: supplementation in neonatal period

**Figure 3:**
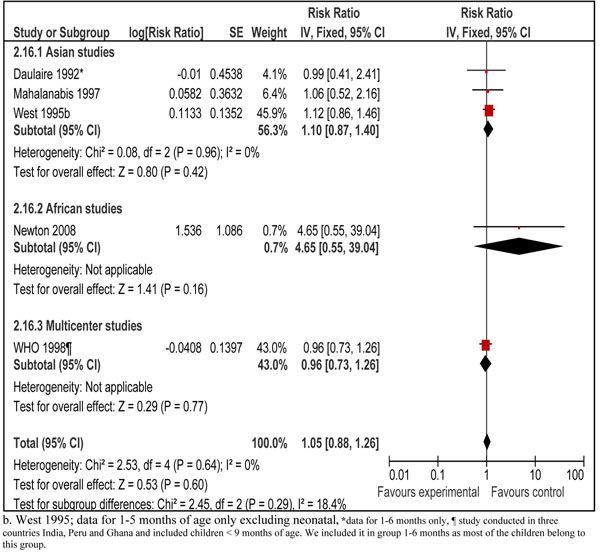
Forest plot for preventive effect of vitamin A supplemenation on all-cause mortality with subgroup analysis for geographical region: supplementation in children 1-6 months of age

**Figure 4:**
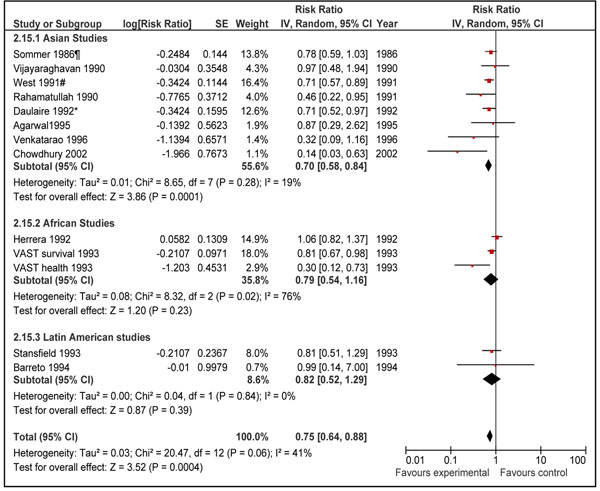
Forest plot for preventive effect of vitamin A supplemenation on all-cause mortality with subgroup analysis for geographical region: supplementation in children 6-59 months of age

Pooled results from all the 21 included studies showed that prophylactic vitamin A supplementation reduced all-cause mortality by 15% [RR 0.85, 95% CI: 0.76-0.94, random model] in children 0-59 months of age (data not shown). We did separate analyses for vitamin A supplementation in neonates, infants 1-6 months and children 6-59 months of age. Data for preventive effect of neonatal vitamin A supplementation was analyzed in two stages: one when infant mortality was measured at 6 months and then that at 12 months of age. Combined results from six studies in neonatal period showed that vitamin A supplementation reduced all-cause mortality by 12% at six months of age [RR 0.88, 95% CI: 0.79-0.98, fixed model]. The sub-group analysis with respect to geographical region for this outcome is also shown which showed that neonatal vitamin A supplementation has a protective effect in Asia but not in Africa (Figure 2). The overall result was, however, not statistically significant at 12 months of age [RR 0.90, 95% CI: 0.56-1.46, random model] (data not shown). In children 1-6 months of age, vitamin A supplementation had no effect on all-cause mortality [RR 1.05; 95 % CI 0.88-1.26, fixed model] (Figure 3). Preventive vitamin A supplementation in children 6-59 months of age reduced all-cause mortality by 25% [RR 0.75, 95% CI: 0.64-0.88, random model], with geographical sub-group analysis shown (Figure 4). This subgroup analysis indicated that preventive vitamin A supplementation had a prominent effect in reducing all-cause mortality in Asia but the results for Africa and Latin America were not statistically significant. Applying a test for subgroup difference, showed no statistically significant difference among the groups (p=0.16). In infants 1-6 months there was no effect in any of the geographical regions. In group 6-59 months of age, five studies included children > 5 years of age and disaggregated data were not available. Excluding these studies from the analysis gives an estimate of 0.72 (95 % CI 0.62-0.83) which is not statistically different form the overall estimate (p=0.36).

#### Disease specific mortality

**Figure 5:**
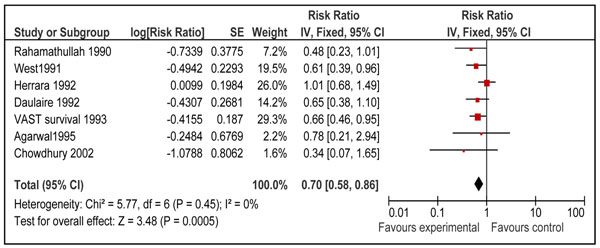
Forest plot for preventive effect of vitamin A supplementation on cause specific mortality of diarrhea: Supplementation in children 6-59 months of age

**Figure 6:**
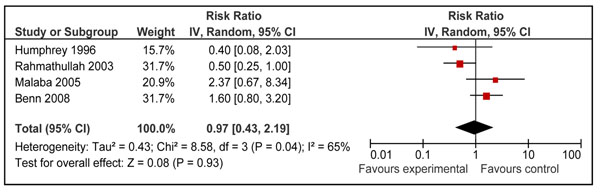
Forest plot for preventive effect of vitamin A supplementation on cause specific mortality of diarrhea: Supplementation in neonatal period

**Figure 7:**
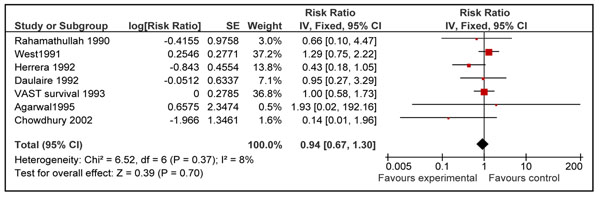
Forest plot for preventive effect of vitamin A supplementation on cause specific mortality of pneumonia: Supplementation in children 6-59 months of age

**Figure 8:**
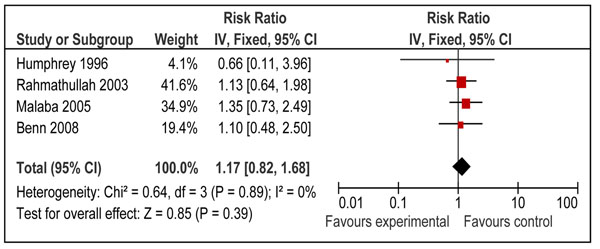
Forest plot for preventive effect of vitamin A supplementation on cause specific mortality pneumonia: Supplementation in neonatal period

Twelve studies reported disease specific mortality of diarrhea and pneumonia [21,23-26,29,31,34-37,40]. Four of these studies included neonates only [31,35-37] and seven included children 6-59 of age [21,23-26,29,34]. Only one study in age group 1-6 months reported cause specific mortality [40]. Pooled results from these studies showed that prophylactic vitamin A supplementation reduces diarrhea specific mortality by 26% [RR 0.74, 95% CI: 0.58-0.93, random model] in children 0-59 months of age. This effect was also significant for supplementation in children 6-59 months of age [RR 0.70, 95% CI: 0.58-0.86, fixed model] (Figure 5), but not on mortality at 12 months for neonatal supplementation [RR 0.97, 95% CI: 0.43-2.19, random model] (Figure 6). One study from group 1-6 months reported a non-significant reduction of 12 % in diarrhea specific mortality [RR 0.88; 95 % CI 0.28-2.81] [40]. Vitamin A supplementation had no overall effect on disease specific mortality of pneumonia [RR 1.05, 95% CI: 0.82-1.33, fixed model] in children 0-59 months of age. A subgroup analysis showed no effect for children 6-59 months [RR 0.94; 95 % CI 0.67-1.30, fixed model] (Figure 7) and neonates [RR 1.17, 95 % CI: 0.82-1.68, fixed model] (Figure 8). One study in group 1-6 months also showed no effect [RR 1.33; 95 % CI 0.37-4.80] [40].

**Figure 9:**
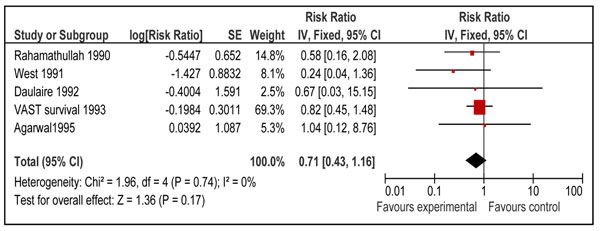
Forest plot for preventive effect of vitamin A supplementation on cause specific mortality of measles: Supplementation in children 6-59 months of age

Disease specific mortality of measles was reported by six studies [21,23,24,26,29,37]. From these studies only one study addressed vitamin A supplementation in neonatal period [37]. Pooled results from these studies showed that vitamin A supplementation reduced measles specific mortality [RR 0.73, 95% CI: 0.47-1.13, fixed model]; however, the results were not statistically significant. Results were similar for children 6-59 months of age [RR 0.71, 95% CI: 0.43-1.16, fixed model] (Figure 9) when mortality results of neonatal study [RR 0.8, 95% CI: 0.3-2.2] were excluded from the analysis. No study reported measles specific mortality in infants 1-6 months of age.

**Figure 10:**
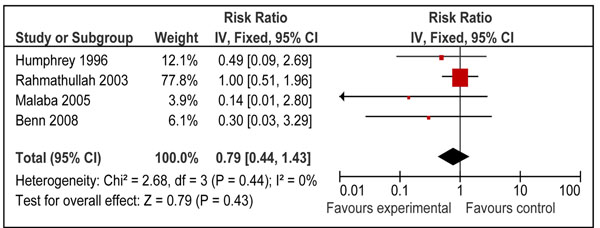
Forest plot for preventive effect of vitamin A supplementation on cause specific mortality of Meningitis: Supplementation in neonatal period

**Figure 11:**
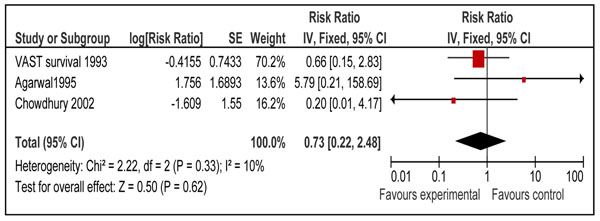
Forest plot for preventive effect of vitamin A supplementation on cause specific mortality of Meningitis: Supplementation in children 6-59 months of age

**Figure 12:**
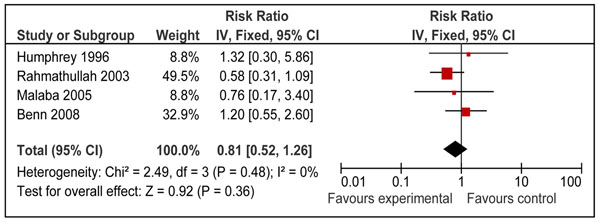
Forest plot for preventive effect of vitamin A supplementation on cause specific mortality of Sepsis: Supplementation in neonatal period

We also looked for effect of vitamin A supplementation on sepsis and meningitis specific mortality. Combined results from eight studies [26,29,31,34-37,40] showed that prophylactic vitamin A supplementation had no significant effect on meningitis specific mortality [RR 0.76, 95% CI: 0.45-1.29, fixed model] in children 0-59 months of age. A subgroup analysis for mortality at 12 months for supplementation in neonates [RR 0.79, 95% CI: 0.44-1.43, fixed model] (Figure 10) and for mortality in children 6-59 months of age [RR 0.73, 95% CI: 0.22-2.48, fixed model] (Figure 11) had similar results. A study from group 1-6 months also showed no significant effect [RR 0.35; 95 % CI 0.01-8.53] on meningitis specific mortality[40]. Four neonatal studies reported sepsis specific mortality at 12 months [31,35-37] and the pooled results showed that vitamin A supplementation had no significant effect on sepsis specific mortality [RR 0.81, 95% CI: 0.52-1.26, fixed model] (Figure 12).

#### Recommendations for LiST model

We followed standardized guidelines to get estimates of efficacy of vitamin A supplementation in reducing diarrhea, pneumonia, measles and meningitis specific mortality, for input to LiST model [9]. In this paper we have presented data for neonates, infants 1-6 months and children 6-59 months of age; however, for the LiST tool, we had focused on generating estimates for efficacy of vitamin A supplementation in children 6-59 months of age. No recommendations for neonatal vitamin A supplementation are being made as neonatal vitamin A supplementation is being evaluated as a separate intervention [17] and randomized controlled trials are currently undergoing in Pakistan, India, Tanzania and Malawi that will provide more evidence on the topic. Also no recommendation is being made for infants 1-6 months because vitamin A supplementation has no effect in this age group as shown previously [18] and in this review.

Recommendations for LiST model are based on qualitative evaluation of pooled estimate and quantitative inferences based in CHERG rules [9]. Additional File 3 summarizes the quality assessment and pooled estimates for disease specific mortality. Vitamin A supplementation reduced diarrhea specific mortality by 30% [95% CI: 14% to 42%] in children 6-59 months of age. The qualitative assessment of the available evidence was that of ‘moderate” level. We recommended the above mentioned estimate for LiST model based on CHERG ‘rule 2’ which states that “***If*** there is high- or moderate-quality evidence of effect on cause-specific mortality…***Then*** use the mortality effect”. The p-value for this estimate was <0.0001, which shows the strength of statistical association.

Pooled estimates for effect of vitamin A supplementation showed a non-significant reduction in pneumonia [RR 0.94 95% CI: 0.67-1.30, p=0.70], measles [RR 0.71, 95% CI: 0.43-1.16, p=0.17] and meningitis [RR 0.73, 95% CI: 0.22-2.48, p=0.62] specific mortality in children 6-59 months of age. The quality grade for all these estimates was that of ‘low’ level. These estimates are not being recommended for LiST model as results were not statistically significant and p value for all the above estimates was more than 0.10. According to CHERG method’s paper a p value of < 0.10 is required for pooled estimate to be considered for inclusion in the LiST tool [9].

## Discussion

The impact of vitamin A supplementation on infant and childhood mortality had been reviewed previously and it has been established that vitamin A has a definite role in reducing all-cause mortality in children older than six months of age [4,5,7,18,42]. A recent trial conducted in India, that is not yet published, showed that vitamin A supplementation has no effect in prevention of mortality in children aged 12-72 months [RR 0.96, 95 % CI: 0.89-1.03] [43]. Although we did not include results of this study in our analysis, as we required full text for GRADE evaluation, combining its results with other studies does not change the results significantly for all-cause mortality in children 6-59 months of age [RR 0.78, 95 % CI: 0.68-0.91, random model].

Preventive vitamin A supplementation in children 6-59 months of age showed a highly significant reduction of 30% (95% CI: 14%-42%) in diarrhea specific mortality. There was no heterogeneity in the pooled data (I^2^=0%). The qualitative assessment of the available evidence according to GRADE criteria was that of ‘moderate’ level. The estimated reduction also corresponds to reduction in all-cause mortality in the same age group (Figure 4) and diarrhea specific mortality reported in other reviews [4,5]. This concludes that reduction of 30 % (95% CI: 14%-42%) is the best estimate of efficacy of vitamin A supplementation in reducing diarrhea specific mortality in children 6-59 months of age.

Despite the non-significant findings in our meta-analysis, an effect of vitamin A supplementation on measles-specific mortality is biologically plausible given the strong beneficial effect of vitamin A for treatment of measles at 200,000 IU for 2 days [RR: 0.40 95% CI 0.19-0.87] [13]. Preventive effect of vitamin A supplementation for measles related mortality may not be as apparent as administration at the onset of disease; as the incidence of disease has decreased at the first place due to large scale measles vaccination and extremely large trials are required to detect a statistically significant difference in measles related mortality. Nevertheless, in the absence of statistically significant data to support an effect, preventive vitamin A supplementation cannot be linked with measles mortality in this edition of the LiST model [9]. Impacts of preventive vitamin A supplementation on other disease specific mortalities like that of meningitis [RR 0.73, 95% CI: 0.22-2.48] and pneumonia [RR 0.94, 95% CI: 0.67-0.1.30] also showed a non-significant reduction in children 6-59 months of age. A review by WHO pneumonia working group also showed that vitamin A supplementation had no beneficial effect on pneumonia related mortality in children more than six months of age [18]. In the same review, data was pooled for all-cause and pneumonia specific mortality in different age groups. The overall results for all-cause mortality in children < 5 years was similar to ours [RR 0.77; 95 % CI 0.71-0.84]. Also there was no significant effect of vitamin A on all-cause and pneumonia specific mortality in infants 0-5 months of age [RR0.97; 95 % CI 0.73-1.29 and 0.88; 95% CI 0.51-1.51 respectively]. Our results for age group 1-6 months also showed no significant effect on all-cause mortality [RR 1.05; 95 % CI 0.88-1.26]. These findings may be attributed to the fact that about 75 % of pneumonia related deaths in pre-school children occur in infants and about 85 % of them occur in infants < 6 months of age [18] and vitamin A has no effect in reducing pneumonia specific mortality as is shown in the above mentioned review [18].

Assessment of protective effect of neonatal vitamin A supplementation on infant mortality has been inconsistent. In a review published in Lancet Under-nutrition Series [7] combining three neonatal vitamin A studies showed a reduction of 20% [95% CI: 4% to 34%] in vitamin A supplemented group compared to control at 6 months of age. The results, however, were not statistically significant at 12 months of age [0.90, 95 % CI: 0.61-1.32]. A review published in BMJ by Gogia et al [8] combined data from six randomized controlled trials also suggested that neonatal vitamin A supplementation has no protective effect on all-cause mortality at 12 months of age [RR 0.92, 95% CI: 0.75-1.12]. We were able to get disintegrated data for all-cause mortality at six months of age from all neonatal studies. Our results showed that neonatal vitamin A supplementation reduced infant mortality by 12% (95 CI: 2% to 21%] at 6 months of age. This implies that neonatal vitamin A supplementation may have more prominent effect in reducing early infant mortality (< 6 months of age) than that at late infancy. As more evidence for efficacy of neonatal vitamin A supplementation will be available from various regions of the world, it may be appropriate to wait for the results of large scale trials in Asia and Africa before recommending this intervention for inclusion in the LiST model.

**Figure 13:**
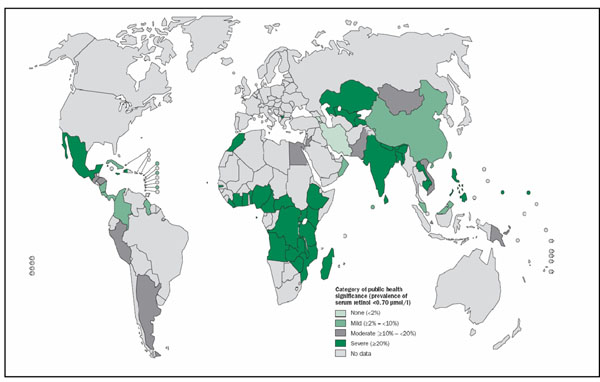
**Distribution of Biochemical vitamin A deficiency (retinol) as a public health problem by country 1995-200 in Preschool-age children**: (Taken from; Global prevalence of vitamin A deficiency in populations at risk 1995–2005. WHO Global Database on Vitamin A Deficiency? Geneva, World Health Organization, 2009)

It is interesting to note that preventive vitamin A supplementation has a differential effect in reducing all-cause mortality in Asia compared to Africa and Latin America (Figures 2 and 4). However the subgroup difference for children 6-59 was not statistically significant (p=0.16). This differential effect has already been described for neonatal vitamin A supplementation [44,45] but has not been noted previously for supplementation in children 6-59 months of age. The exact reasons for these differential effects are not known however one explanation could be that according to WHO [19], the highest proportions of pre-school age children with biochemical vitamin A deficiency (serum retinol <0.70 μmol/l), live in South-East Asia (Figure 13). Africa on the other hand has the second highest prevalence of biochemical vitamin A deficiency. It is important to note that two of three studies conducted in Africa in children 6-59 showed a statistically significant effect in reducing all-cause mortality. The confidence interval of pooled relative risk for Africa was not far above unity. This showed that there may be a protective effect in Africa but not as prominent as that in Asia. In any case, there is not sufficient evidence to decide in favor or against of a true differential effect for vitamin A supplementation in Asia vs. Africa and Latin America in children 6-59 months of age.

The dose of vitamin A supplementation in most of the included studies was 200 000 IU every 4 or 6 months. It has been suggested previously that this regimen of vitamin A supplementation may just be marginally sufficient to keep adequate stores of vitamin A in the body [5]. One of the included studies that used weekly supplementation showed more prominent effect in reducing all-cause mortality compared to other studies [21]. In any case the most important inference to make is that it is the improvement of vitamin A status rather than the method of improving it that makes it the important determinant in reducing all-cause mortality.

Vitamin A supplementation seems to be a relatively safe intervention. Few children may suffer mild adverse effects with the standard dosage of vitamin A supplementation; however, they are rare and transient. The most commonly reported side effects include irritability, loose stools, headache, fever, nausea, vomiting and bulging fontanel (in neonates) [46-48]. Although we did not specifically pool results for different side effects profiles; however, results from previous reviews showed that there is no significant increased risk of any adverse effect. In the review by Gogia et al, pooled results from the included studies showed that vitamin A supplementation in neonates did not significantly increase risk of bulging fontanel, vomiting, irritability, diarrhea, or fever [8].

Our review has several limitations. Three of the included studies had comparison groups that received no placebo [20,24,39]. This may bias the results through the “Hawthorne effect”[49]. We were unable to identify any significant predictor of substantial heterogeneity for all-cause and disease specific mortalities. There may be factors, not examined by us, that might explain the observed differences. For example, effects of micronutrient supplementation might be different between boys and girls [42,50]. In the review by Gogia et al. pooled results from four neonatal studies showed that the risk of mortality was lower among boys [RR 0.77, 95 % CI: 0.59, 1.01) than girls (RR 0.93, 95 % CI: 0.73, 1.17) [8]. These divergent results for neonatal vitamin A supplementation might be explained by differences in vaccination intensity as vitamin A supplementation has been shown to interact negatively with DPT vaccination in girls [37,51]. Other possible important factors contributing to heterogeneity may include feeding practices of participants, dosage and number of vitamin A supplementation, vaccination status, maternal vitamin A status and HIV prevalence [12,45,52,53].

In conclusion, vitamin A supplementation has a definite role in reducing all-cause and diarrhea specific mortality in children 6-59 months of age. Impacts for measles and meningitis specific mortality, however, showed no effect. There is also no role of vitamin A supplementation for prevention of pneumonia specific mortality. While there is a suggestion of benefit of neonatal vitamin A supplementation on mortality at 6 months of age, this observation must be verified in other large scale studies in varied geographic regions and await further information from the four large scale trials which are underway.

## Competing interests

We do not have any financial or non-financial competing interests for this review.

## Authors' contributions

Professor Zulfiqar A Bhutta developed the review parameters and secured support. Dr Aamer Imdad and Christopher Sudfeld undertook the literature search, data extraction and analysis supervised by Professor Bhutta. Dr Mohammad Yawar Yakoob double abstracted the data and critically reviewed and modified the manuscript in addition to Dr Batool Haider and Professor Robert E Black and Professor Bhutta

## Supplementary Material

Additional File 1 Characteristics of included studies Click here for file

Additional File 2 Risk of bias for the included studies according to the latest recommendations of the Cochrane Handbook Click here for file

Additional File 3 Quality assessment of trials of vitamin A supplementation assessing effect on cause specific mortality in infants and children Click here for file
